# Effect of Successive Bleaching Treatments Followed by Staining on Color, Whiteness, and Translucency of Polymer‐Based CAD‐CAM Materials

**DOI:** 10.1111/jerd.70168

**Published:** 2026-04-26

**Authors:** Alejandro Cardenas Ramos, Bruno Arruda Mascaro, José Maurício dos Santos Nunes Reis, Lívia Nordi Dovigo, María M. Pérez, Renata Garcia Fonseca

**Affiliations:** ^1^ Department of Dental Materials and Prosthodontics, School of Dentistry São Paulo State University (UNESP) Araraquara São Paulo Brazil; ^2^ Department of Social Dentistry, School of Dentistry São Paulo State University (UNESP) Araraquara São Paulo Brazil; ^3^ Department of Optics, Faculty of Science University of Granada Granada Spain

**Keywords:** bleaching, CAD/CAM materials, color, translucency, whiteness

## Abstract

**Objective:**

To evaluate differences in color, whiteness, and translucency of CAD‐CAM materials after successive bleaching treatments and coffee immersion.

**Materials and Methods:**

Lava Ultimate (LU) and VITA Enamic (VE) were divided into four groups (*n* = 12): untreated (control), 35% hydrogen peroxide (WH), 10% carbamide peroxide (OP), or WH followed by OP (WHOP). Color measurements were taken at baseline (*T*_0_) and after three consecutive bleaching sessions, each followed by coffee exposure (*T*_1_, *T*_2_, and *T*_3_). Differences in color (Δ*E*_00_), whiteness (ΔWI_D_), and translucency (ΔRTP_00_) were statistically analyzed for each material using two‐way ANOVA and Bonferroni's tests and interpreted based on their respective perceptibility and acceptability thresholds. Surface topography was assessed by scanning electron microscopy.

**Results:**

For LU, there were no differences in Δ*E*_00_ and ΔWI_D_ among bleaching treatments. The second and third sessions increased Δ*E*_00_ and ΔWI_D_ values. The WH showed the greatest imperceptible ΔRTP_00_. In VE, no treatment increased Δ*E*_00_, and only OP showed higher ΔE_00_ in *T*_3_–*T*_0_ compared with *T*_1_–*T*_0_, but it was still imperceptible. All treatments decreased ΔWI_D_ in *T*_2_–*T*_0_ and *T*_3_–*T*_0_, with no significant differences in ΔRTP_00_ compared with control. Except for WHOP, there were no statistical differences among sessions for ΔRTP_00_. Only WH in VE did not result in topographic changes.

**Conclusions:**

Successive bleaching treatments and coffee immersion resulted in imperceptible or deemed acceptable color, whiteness, and translucency changes of LU and VE.

**Clinical Significance:**

Despite the surface topographic changes, all the bleaching methods tested in up to three sessions each followed by exposure to coffee did not adversely affect the esthetic durability of Lava Ultimate and VITA Enamic CAD‐CAM materials.

## 1 Introduction

Since monolithic materials of different compositions have common indications, such as laminate veneer, inlays, and onlays, some aspects must be considered when selecting them to obtain esthetic restorations. It is known that these materials, mainly the resin‐based and hybrid ones, can undergo changes in their esthetic properties when exposed to stains from diets [1, 2, 3, 4, 5, 6, 7]. When these changes exceed the color acceptability threshold established in the literature [8, 9], which is not so uncommon, some clinical procedure must be performed to avoid replacing the restoration.

At‐home and in‐office bleaching has been shown to be effective in the partial or total recovery of color, whiteness, and/or translucency of these resin‐containing materials [4, 10, 11, 12, 13, 14]. However, it is known that, like polishing [15, 16], bleaching [17, 18, 19, 20, 21, 22, 23] can modify the surface texture of these materials. One question that arises is whether such changes would make the materials even more susceptible to color, whiteness, and translucency variations when again exposed to extrinsic pigments. Little has been studied about this scenario [12, 18, 24, 25, 26]. Among these studies, those that evaluated the effect of hydrogen peroxide as an in‐office bleaching agent used a concentration of 40% [12, 25, 26], which caused an adverse effect on some of the esthetic properties in resin‐based composite CAD‐CAM monolithic materials. Similarly, studies evaluating carbamide peroxide found adverse effects on the esthetic properties of VITA Enamic after using a low concentration of this at‐home bleaching agent but for a considerable exposure time (CP 10%; 10 h/day for 14 days) [24] or a higher concentration but for a shorter exposure time (16%; 6 h/day for 7 days) [18]. No study about susceptibility to staining after bleaching evaluated a low concentration of carbamide peroxide in a short exposure time, which was also effective in recovering the esthetic properties of resin‐based composite and polymer‐infiltrated ceramic network CAD‐CAM monolithic materials [11].

Another concern is the fact that it is not uncommon for patients to undergo bleaching more than once, leading us to question how CAD‐CAM materials would behave if bleached as frequently as teeth. The only study [24] that evaluated the effect of successive bleaching treatments subsequently followed by staining on the esthetic properties of Lava Ultimate, VITA Enamic, IPS Empress CAD, and IPS e.max CAD materials employed at‐home bleaching (CP 10%; 10 h/day for 14 days), reporting adverse effects due to the high exposure time.

In addition, the combination of bleaching treatments, that is, in‐office bleaching followed by at‐home bleaching, has been widely performed in clinical practice. To the best of the authors' knowledge, no studies have specifically investigated the combined effects of in‐office and at‐home bleaching protocols and coffee exposure on the esthetic properties of monolithic CAD‐CAM materials, not even in a single session. Given the above, the purpose of the present study was to evaluate the effect of different bleaching treatments in up to three sessions, each followed by exposure to coffee, on susceptibility to color, whiteness, and translucency variations in resin‐based composite and polymer‐infiltrated ceramic network CAD‐CAM monolithic materials. The research hypotheses were that (H1) the different bleaching treatments, and that (H2) the successive bleaching sessions would influence the changes in color, whiteness, and translucency in each material.

## 2 Materials and Methods

### 2.1 Specimen Preparation and Study Design

**FIGURE 1:**
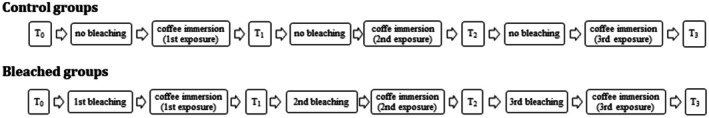
Methodological scheme of the control and experimental groups.

The materials evaluated are listed in Table 1. The sample size calculation by G*Power (v.3.1.9.7; Heinrich‐Heine‐Universität Düsseldorf), considering an *F*‐test for repeated measures ANOVA, within‐between interaction, a significance level of *α* = 0.05 and power of *β* = 0.20 determined a minimum of 12 specimens per group [27]. CAD/CAM blocks were milled into *Ø*10.00‐mm cylinders and sliced into disks by a precision saw (IsoMet 1000; Buehler, Lake Bluff, IL, USA). Disks were polished with silicon carbide papers (600‐, 1200‐, 1500‐grit) under water irrigation, resulting in 1.00 ± 0.02 mm thickness, and randomly allocated into four groups (*N* = 48/material). The treatments of the specimens were: (1) untreated (control), (2) in‐office bleaching with 35% hydrogen peroxide (WH), (3) at‐home bleaching with 10% carbamide peroxide (OP), and (4) a combined protocol (WHOP). Figure 1 displays the methodological scheme of the control and experimental groups.

**TABLE 1:** CAD‐CAM monolithic materials and bleaching gels evaluated.

Material	Classification	Composition	Manufacturer
Lava Ultimate A2‐HT (LU)	Resin nanoceramic	Agglomerated nanoparticles of silica and zirconia (80% by weight), highly cross‐linked polymer matrix composed of Bis‐GMA, UDMA, Bis‐EMA, and TEGDMA (20% by weight). Particle sizes: 20‐nm silica particles, 4‐ to 11‐nm zirconia particles	3M ESPE
VITA Enamic 2M2‐HT (VE)	Polymer‐infiltrated ceramic network	Fine structure feldspathic ceramic (86% by weight), resin polymer composed of UDMA and TEGDMA (14% by weight)	VITA Zahnfabrik
Whiteness HP Blue (WH)	In‐office bleaching agent	Hydrogen peroxide 35% Inactive ingredients: thickeners, inert pigment, neutralizing agents, calcium gluconate, glycol, and purified water	FGM
Opalescence PF (OP)	At‐home bleaching agent	Carbamide peroxide 10%, 40%–60% glycerol, 9%–11% hydrogen peroxide—urea, 1%–10% polyacrylic acid, 1%–10% polyethylene glycol, 1%–5% sodium hydroxide	Ultradent

### 2.2 Bleaching and Coffee Immersion Protocols

Three sessions were carried out for each bleaching modality. In‐office bleaching was performed with 35% hydrogen peroxide (Whiteness HP Blue, FGM, Dentscare) and each session consisted of one single 45‐min application. The specimens were then washed and stored in distilled water in an incubator at 37°C. At‐home bleaching was performed with 10% carbamide peroxide (Opalescence PF; Ultradent Products), and each session consisted of application of the bleaching gel for 6 h/day for 7 days [12]. After each 6‐h application, the specimens were washed and stored in distilled water in an incubator at 37°C until the next application, which occurred after 24 h. Regarding the combined protocol (WHOP), in‐office bleaching was followed by at‐home bleaching, according to the same procedures described in individual bleaching. There was no time interval between the protocols. For the three modalities, the bleaching agents were applied to the top surface of the specimens at an approximately 1 mm thickness with a microbrush. The specimens were kept in an incubator at 37°C during bleaching, simulating the temperature of the oral cavity.

For the coffee exposure, a coffee (Ristretto; Nespresso, Lausanne, Switzerland) solution was prepared daily in the long mode of a machine (Essenza Mini, Nespresso; Nestlé). Toward simulating a 1‐year exposure, the specimens were immersed for 30 min daily for 36.5 days, considering an intake frequency of three cups per day and an exposure time of 60 s per cup [28]. After each coffee exposure, the specimens were circumferentially brushed 10 times under running water and then stored in deionized water until the next exposure.

The sequence of bleaching followed by coffee exposure was designed to simulate post‐bleaching exposure to chromogenic agents and to evaluate whether bleaching‐induced surface changes that could influence subsequent discoloration.

Baseline analyses (*T*_0_) were performed after the specimens had been bleached or not and subsequently immersed in coffee. This process was repeated three more times and new analyses were conducted (*T*_1_, *T*_2_, and *T*_3_).

### 2.3 Color, Whiteness, and Relative Translucency Parameter

Color measurements of the specimens were taken using a calibrated spectrophotometer (CM‐2600d, Konica Minolta, Osaka, Japan) coupled with software (OnColor V5, CyberChrome, Beech Island, SC, USA) over white (L* = 99.46, a* = −0.12, and b* = −0.04) and black (L* = 17.80, a* = −0.13, and b* = −0.65) backgrounds (CM‐A145 calibration plate, Konica Minolta). CIE L*a*b* color coordinates values were obtained in triplicate using D65 Standard Illuminant, CIE di/8**°** illuminating/measuring geometry, and CIE 2**°** Standard Observer, and the data were averaged. Differences in color (Δ*E*_00_), whiteness (ΔWI_D_), and relative translucency parameter (ΔRTP_00_) were determined based on the L*, a*, and b* values between *T*_1_ and *T*_0_, *T*_2_ and *T*_0_, and *T*_3_ and *T*_0_.

The L*, a*, and b* mean values from each time interval were computationally converted to CIEDE2000 C* and H* coordinates to calculate color difference with the following equation (1:1:1) [29]:

$$∆E_{00}={\left[{\left(\frac{∆L^{\prime }}{k_{L}S_{L}}\right)}^{2}+{\left(\frac{∆C^{\prime }}{k_{C}S_{C}}\right)}^{2}+{\left(\frac{∆H^{\prime }}{k_{H}S_{H}}\right)}^{2}+R_{T}\left(\frac{∆C^{\prime }}{k_{C}S_{C}}\right)\left(\frac{∆H^{\prime }}{k_{H}S_{H}}\right)\right]}^{\frac{1}{2}}$$

where Δ*L* ´, Δ*C* ´, and Δ*H* ´ refer to lightness, chroma, and hue differences between color measurements, respectively. *k*_*L*_, *k*_*C*_, and *k*_*H*_ are the parametric factors for the conditions and illuminating influence set at 1. *R*_T_ (rotation function) accounts for the interaction of hue and chroma differences in the blue region, and *S*_*L*_, *S*_*C*_, and *S*_*H*_ are the weighting functions for the color difference adjustment, considering the location variation of L*, a*, and b* coordinates [9, 29]. Metric discontinuities due to mean hue computation and hue difference computation were considered in the Δ*E*_00_ calculation [30].

The interpretation of Δ*E*_00_ was based on published data for 50:50% color perceptibility (PT_00_ = 0.8) and acceptability (AT_00_ = 1.8) thresholds [8] standardized by the CIE 015:2018 [9]. Moreover, the following interpretation of color differences over AT_00_ was adopted: moderately unacceptable (1.8 < Δ*E*_00_ ≤ 3.6), clearly unacceptable (3.6 < Δ*E*_00_ ≤ 5.4), and extremely unacceptable (Δ*E*_00_ > 5.4) [31].

Whiteness index for dentistry (WI_D_) was used for the evaluation of whiteness and calculated by the following equation [32]:

$${\mathrm{WI}}_{D}=0.511L^{*}-2.320a^{*}-1.100b^{*}$$

whiteness differences were calculated by $∆{\mathrm{WI}}_{D}=∆{\mathrm{WI}}_{D\;\left(\text{final}\right)}-∆{\mathrm{WI}}_{D\;\left(\text{initial}\right)}$.

The interpretation of ΔWI_D_ was based on published data for 50%:50% whiteness perceptibility (WPT = 0.72) and acceptability (WAT = 2.62) thresholds [33].

Relative translucency parameter (RTP) was calculated by CIEDE2000 (1:1:1) color difference formula [34]:

$$\begin{array}{c} {\mathrm{RTP}}_{00}=[{\left(\frac{L\prime_{B}-L\prime_{W}}{k_{L}S_{L}}\right)}^{2}+{\left(\frac{C\prime_{B}-C\prime_{W}}{k_{C}S_{C}}\right)}^{2}+{\left(\frac{H\prime_{B}-H\prime_{W}}{k_{H}S_{H}}\right)}^{2}\\ \;{+R_{T}\left(\frac{C\prime_{B}-C\prime_{W}}{k_{C}S_{C}}\right)\left(\frac{H\prime_{B}-H\prime_{W}}{k_{H}S_{H}}\right)]}^{\frac{1}{2}} \end{array}$$

where *B* and *W* refer to specimens over black and white backgrounds, respectively.

Translucency differences were calculated by $∆{\mathrm{RTP}}_{00}={\mathrm{RTP}}_{00\;\left(\text{final}\right)}-{\mathrm{RTP}}_{00\;\left(\text{initial}\;\right)}$.

ΔRTP_00_ was analyzed using the 50%:50% translucency perceptibility (TPT_00_ = 0.62) and acceptability (TAT_00_ = 2.62) thresholds [34].

### 2.4 Surface Topography

As SEM analysis is qualitative and descriptive in nature, a formal sample size calculation was not performed. To assess variations in surface topography, additional specimens (*n* = 2/group) were prepared, gold‐sputtered, and multiple areas were examined under a high‐resolution field emission scanning electron microscope (FIB FEI, Helios Nanolab 6001, Hillsboro, Oregon, USA) at 25,000× magnification and 2.0 kV accelerating voltage.

### 2.5 Statistical Analysis

Data were evaluated regarding the assumption of normality (Shapiro–Wilk test; *p* ≥ 0.05) and sphericity (Mauchly test). Δ*E*_00_, ΔWI_D_, and ΔRTP_00_ data were analyzed for each material and esthetic property by mixed repeated measures ANOVA (paired factor: Δ*T*_1_–*T*_0_, Δ*T*_2_–*T*_0_, and Δ*T*_3_–*T*_0_; independent factor: treatment), with lower bound correction in cases where sphericity was not assumed. When the ANOVA indicated statistically significant effects, a multiple comparison analysis was performed using the Bonferroni test (for the main factors or for the interaction, depending on what was indicated in the ANOVA). The significance level was set at *α* = 0.05. IBM SPSS v23.0 (IBM, Armonk, NY, USA) was used for statistical analyses.

## 3 Results

Table 2 shows the results of LU. In the three sessions, there was no statistical difference in Δ*E*_00_ and ΔWI_D_ among the treatments. In all treatments, Δ*E*_00_ values were lower in *T*_1_–*T*_0_ than in *T*_2_–*T*_0_ and *T*_3_–*T*_0_ (*p* ≤ 0.001). In all groups, the highest negative values of ΔWI_D_ were observed in *T*_2_–*T*_0_ and *T*_3_–*T*_0_ compared with *T*_1_–*T*_0_ (*p* ≤ 0.001), indicating a decrease in whiteness. All treatments provided acceptable Δ*E*_00_ and ΔWI_D_, except for WHOP in *T*_1_–*T*_0_, where Δ*E*_00_ was imperceptible. In all three sessions, WH provided higher negative ΔRTP_00_ values, indicating reduced translucency in comparison with the control (*p* < 0.05). No treatment showed a significant difference in ΔRTP_00_ among the sessions. All treatments provided imperceptible ΔRTP_00_.

**TABLE 2:** ∆*E*_00_, ∆WI_D_, and ∆RTP_00_ mean values of Lava Ultimate according to treatment and time interval.

	∆*E*_00_			∆WI_D_			∆RTP_00_		
	*T*_1_–*T*_0_	*T*_2_–*T*_0_	*T*_3_–*T*_0_	*T*_1_–*T*_0_	*T*_2_–*T*_0_	*T*_3_–*T*_0_	*T*_1_–*T*_0_	*T*_2_–*T*_0_	*T*_3_–*T*_0_
Control	*0.94 ± 0.35 Ab	*1.42 ± 0.52 Aa	*1.35 ± 0.38 Aa	*−1.12 ± 0.48 Aa	*‐1.64 ± 0.77 Ab	*‐1.72 ± 0.49 Ab	−0.11 ± 0.27 Aa	−0.17 ± 0.58 Aa	−0.31 ± 0.45 Aa
WH	*0.84 ± 0.19 Ab	*1.10 ± 0.26 Aa	*1.24 ± 0.45 Aa	*−1.29 ± 0.59 Aa	*−1.60 ± 0.51 Ab	*−1.71 ± 0.45 Ab	−0.46 ± 0.30 Ba	−0.50 ± 0.24 Ba	−0.48 ± 0.28 Ba
OP	*1.06 ± 0.37 Ab	*1.45 ± 0.45 Aa	*1.71 ± 0.55 Aa	*−1.33 ± 0.53 Aa	**−1.96 ± 0.63 Ab	**−2.11 ± 0.61 Ab	−0.22 ± 0.16 ABa	−0.24 ± 0.14 ABa	−0.35 ± 0.28 ABa
WHOP	0.74 ± 0.30 Ab	*1.13 ± 0.36 Aa	*1.28 ± 0.38 Aa	*−1.09 ± 0.47 Aa	*−1.48 ± 0.38 Ab	*−1.69 ± 0.41 Ab	−0.20 ± 0.19 ABa	−0.20 ± 0.25 ABa	−0.26 ± 0.17 ABa

*Note:* ∆*E*_00_: mixed repeated measures ANOVA (ANOVA_treatment_: *p* = 0.051; ANOVA_time interval_: *p* < 0.001; ANOVA_interaction_: *p* = 0.140; Bonferroni: *p* ≤ 0.001). Δ*E*_00_ ≤ 0.8 (imperceptible), *0.8 < Δ*E*_00_ ≤ 1.8 (acceptable), **1.8 < Δ*E*_00_ ≤ 3.6 (moderately unacceptable), ***3.6 < Δ*E*_00_ ≤ 5.4 (clearly unacceptable). ∆WI_D_: mixed repeated measures ANOVA (ANOVA_treatment_: *p* = 0.190; ANOVA_time interval_: *p* < 0.001; ANOVA_interaction_: *p* = 0.570; Bonferroni: *p* ≤ 0.001). Negative ∆W_ID_ means decreased whiteness compared to that of the baseline. ∆WI_D_ ≤ 0.72 (imperceptible), *0.72 < ∆WI_D_ ≤ 2.62 (acceptable), and **∆WI_D_ > 2.62 (unacceptable). ∆RTP_00_: mixed repeated measures ANOVA (ANOVA_treatment_: *p* = 0.030; ANOVA_time interval_: *p* = 0.062; ANOVA_interaction_: *p* = 0.802; Bonferroni: *p* ≤ 0.039). Negative ∆RTP_00_ means decreased translucency compared to that of the baseline. ∆RTP_00_ ≤ 0.62 (imperceptible), *0.62 < ∆RTP_00_ ≤ 2.62 (acceptable), and **∆RTP_00_ > 2.62 (unacceptable). In columns, different capital letters (A,B) indicate significant differences among the treatments. In lines, different lowercase letters (a,b) indicate significant differences among the bleaching sessions within each optical property.

Abbreviations: OP, Opalescence PF; WH, Whiteness HP Blue; WHOP, Whiteness HP Blue followed by Opalescence PF.

Table 3 shows the results of VE. In all sessions, there was no difference in Δ*E*_00_ among the treatments, except for WH in *T*_3_–*T*_0_, which decreased Δ*E*_00_ when compared with the control (*p* < 0.05). Except for OP, whose Δ*E*_00_ was higher in *T*_3_–*T*_0_ than in *T*_1_–*T*_0_ (*p* ≤ 0.001), there was no difference among the sessions in the other treatments. All groups showed imperceptible Δ*E*_00_. Regarding ΔWI_D_, no treatment differed from the control in *T*_1_–*T*_0_. In *T*_2_–*T*_0_ and *T*_3_–*T*_0_, all treatments reduced ΔWI_D_ (*p* ≤ 0.015), promoting an increase in whiteness compared to the control. In the three sessions, the ΔWI_D_ of OP and WHOP was imperceptible, while in the control and WH it was acceptable. In all sessions, the ΔRTP_00_ of the treatments did not differ from the control. However, in *T*_3_–*T*_0_, the ΔRTP_00_ of OP and WHOP was acceptable, whereas in the control and WH, it was imperceptible. There was no difference among the sessions, except for WHOP, whose ΔRTP_00_ was higher in *T*_3_–*T*_0_ than in *T*_1_–*T*_0_ (*p* ≤ 0.001), with a reduction in translucency.

**TABLE 3:** ∆*E*_00_, ∆WI_D_, and ∆RTP_00_ mean values of VITA Enamic according to treatment and time interval.

	∆*E*_00_			∆WI_D_			∆RTP_00_		
	*T*_1_–*T*_0_	*T*_2_–*T*_0_	*T*_3_–*T*_0_	*T*_1_–*T*_0_	*T*_2_–*T*_0_	*T*_3_–*T*_0_	*T*_1_–*T*_0_	*T*_2_–*T*_0_	*T*_3_–*T*_0_
Control	0.47 ± 0.08 Aa	0.66 ± 0.08 Aa	0.75 ± 0.08 Aa	−0.74 ± 0.12 ABa	*−1.10 ± 0.14 Cb	*−1.22 ± 0.13 Cb	−0.18 ± 0.10 Aa	−0.46 ± 0.17 Aa	−0.46 ± 0.14 ABa
WH	0.51 ± 0.10 Aa	0.43 ± 0.15 Aa	0.44 ± 0.13 Ba	*−0.81 ± 0.18 Ba	*−0.73 ± 0.22 Ba	*−0.73 ± 0.21 Ba	−0.24 ± 0.16 Aa	−0.37 ± 0.18 Aa	−0.37 ± 0.18 Aa
OP	0.38 ± 0.15 Ab	0.47 ± 0.26 Aab	0.75 ± 0.35 Aa	−0.44 ± 0.19 Ab	0.11 ± 0.39 Aa	0.42 ± 0.35 Aa	−0.39 ± 0.42 Aa	−0.56 ± 0.37 Aa	*−0.78 ± 0.46 ABa
WHOP	0.38 ± 0.14 Aa	0.38 ± 0.26 Aa	0.61 ± 0.37 ABa	−0.53 ± 0.22 ABb	0.00 ± 0.18 Aa	0.20 ± 0.36 Aa	−0.17 ± 0.24 Aa	−0.49 ± 0.36 Aab	*−0.87 ± 0.48 Bb

*Note:* ∆*E*_00_: mixed repeated measures ANOVA (ANOVA_treatment_: *p* = 0.023; ANOVA_time interval_: *p* < 0.001; ANOVA_interaction_: *p* = 0.011; Bonferroni: *p* ≤ 0.032). Δ*E*_00_ ≤ 0.8 (imperceptible), *0.8 < Δ*E*_00_ ≤ 1.8 (acceptable), **1.8 < Δ*E*_00_ ≤ 3.6 (moderately unacceptable), ***3.6 < Δ*E*_00_ ≤ 5.4 (clearly unacceptable). ∆WI_D_: mixed repeated measures ANOVA (ANOVA_treatment_: *p* < 0.001; ANOVA_time interval_: *p* < 0.001; ANOVA_interaction_: *p* < 0.001; Bonferroni: *p* ≤ 0.022). Negative ∆W_ID_ means decreased whiteness compared to that of the baseline. ∆WI_D_ ≤ 0.72 (imperceptible), *0.72 < ∆WI_D_ ≤ 2.62 (acceptable), and **∆WI_D_ > 2.62 (unacceptable). ∆RTP_00_: mixed repeated measures ANOVA (ANOVA_treatment_: *p* = 0.070; ANOVA_time interval_: *p* < 0.001; ANOVA_interaction_: *p* = 0.002; Bonferroni: *p* ≤ 0.006). Negative ∆RTP_00_ means decreased translucency compared to that of the baseline. ∆RTP_00_ ≤ 0.62 (imperceptible), *0.62 < ∆RTP_00_ ≤ 2.62 (acceptable), and **∆RTP_00_ > 2.62 (unacceptable). In columns, different capital letters (A,B,C) indicate significant differences among the treatments. In lines, different lowercase letters (a,b) indicate significant differences among the bleaching sessions within each optical property.

Abbreviations: OP, Opalescence PF; WH, Whiteness HP Blue; WHOP, Whiteness HP Blue followed by Opalescence PF.

**FIGURE 2:**
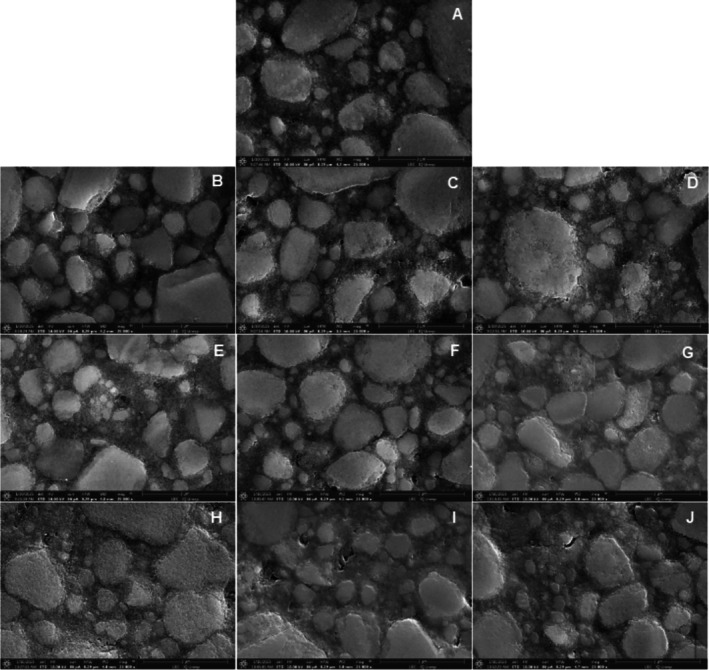
Scanning electron microscope images (original magnification ×25,000) of Lava Ultimate. (A) control group; (B–D) Whiteness HP Blue (WH) first, second, and third sessions; (E–G) Opalescence PF (OP) first, second, and third sessions; (H–J) combined protocol (WHOP) first, second, and third sessions.

**FIGURE 3:**
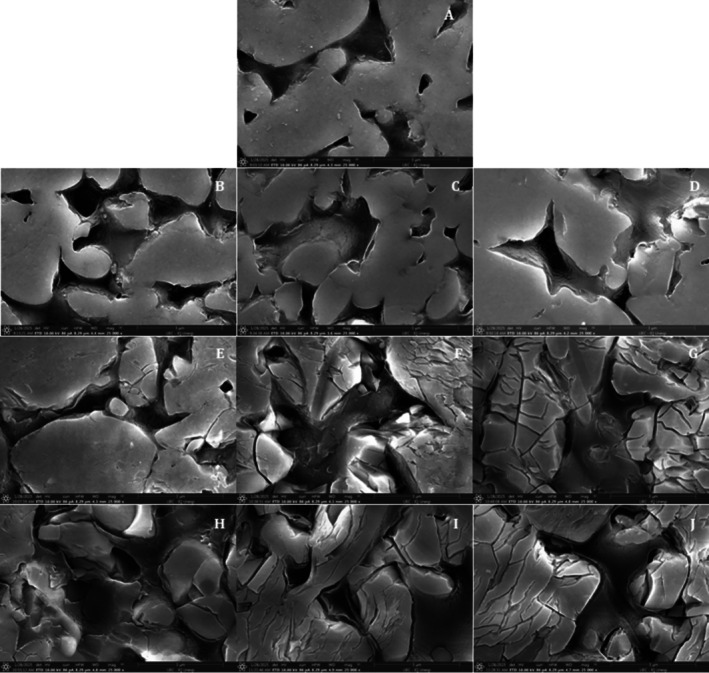
Scanning electron microscope images (original magnification ×25,000) of VITA Enamic. (A) control group; (B–D) Whiteness HP Blue (WH) first, second, and third sessions; (E–G) Opalescence PF (OP) first, second, and third sessions; (H–J) combined protocol (WHOP) first, second, and third sessions.

Figures 2 and 3 display the SEM images. In LU, WH caused progressive degradation of the resin matrix and particles. The OP led to surface smoothing, especially after the 3rd session, whereas WHOP caused a degradation of the matrix in the 1st session followed by smoothing in the other sessions. In VE, WH did not promote topographic changes, whereas OP and WHOP progressively degraded its ceramic portion, leading to microcracks.

## 4 Discussion

This study evaluated how different bleaching treatments, performed in up to three sessions, with each followed by immersion in coffee, affect changes in color, whiteness, and translucency in two polymer‐based CAD‐CAM materials. The hypotheses were accepted, as the different treatments and successive sessions influenced the susceptibility to color, whiteness, and translucency variations in both materials.

Only five related studies, that is, those that performed bleaching followed by staining, were found [12, 17, 22, 24, 25]. Of these, only four [12, 17, 22, 24] evaluated one or more materials used in the present study. Furthermore, only one of them [22] evaluated successive sessions and none evaluated the combination of bleaching treatments.

In the three sessions, no treatment affected the susceptibility to color and whiteness variations of LU, indicating that the increase in the variation of these properties from *T*_1_–*T*_0_ to *T*_2_–*T*_0_ and *T*_3_–*T*_0_ was the result of the accumulation of coffee stain deposition, and only WH increased the susceptibility to translucency variations, which did not vary among sessions, regardless of the treatment. Using a slightly higher hydrogen peroxide concentration and exposure time (HP 40% 3 × 20 min) than those in the present study, a similar study [12] also detected topographic changes in LU, without compromising susceptibility to color and whiteness variations, but with increased susceptibility to translucency variations, with a reduction in this property, which is in line with our findings. In a previous study [20], when evaluating the same bleaching gel and the same exposure protocol [12], the authors also found topographic changes in LU, but its roughness was not affected in the three successive bleaching sessions. This same behavior, that is, topographic changes without influence on roughness, was observed in a parallel study [23] with exactly the same bleaching modalities and sessions as those evaluated in the present study. It seems that translucency was more sensitive to changes in topography than color and whiteness, considering that surfaces with greater texture changes, such as those promoted by WH, lead to greater light dispersion and consequent reduction in translucency. In contrast to WH, the other bleaching treatments tended to promote surface smoothing. This difference in the effect of WH and OP on the topography of LU was also observed in one study [14]. However, this reduction in translucency was imperceptible and, as like in the other treatments, was not progressive. A previous study [18] also did not detect any influence on the susceptibility to whiteness variation of LU, but reported an increase in its susceptibility to color variation, which was possibly the result of the considerably longer exposure time, despite the lower concentration of hydrogen peroxide (HP 6% 1 h/day for 10 days). Regarding carbamide peroxide, despite the clear degradation of the organic matrix observed in an earlier study [24], the authors also found no influence of 10% CP on the susceptibility to variations in translucency or an increase in this property among the sessions, which agrees with our findings. However, the authors [24] reported an increase in susceptibility to variations in color and whiteness from the second session onwards, which may have been due to the longer exposure time used in this study, that is, 10 h/day for 14 days, which justifies the difference between the results. Using an exposure time similar to that of the present study, but with a higher concentration of bleach gel (CP 15%), a similar study [18] also did not observe an increase in susceptibility to color and whiteness variations. As for WHOP, no studies were found that could serve as a comparison parameter for any of the materials evaluated in the present study. However, despite being a combination of bleaching treatments, which could have a greater degree of aggressiveness, such treatment promoted topographic changes, as did the other treatments, but without affecting the susceptibility to color, whiteness, and translucency variations in LU. Based on the findings, although bleaching treatments conducted in up to three sessions may be considered safe with regard to the evaluated esthetic properties, the topographic alterations observed across all protocols may affect other properties and should be carefully taken into account.

As with LU, no treatment adversely affected the susceptibility to color and whiteness variations of VE, despite the notable degradation of the ceramic portion by OP and WHOP, as also reported in a previous study [14]. On the contrary, WH reduced the susceptibility to color variation in *T*_3_–*T*_0_ and whiteness variation in *T*_2_–*T*_0_ and *T*_3_–*T*_0_, however, without altering the classification related to the thresholds, whereas OP and WHOP not only reduced the susceptibility to whiteness variation in *T*_2_–*T*_0_ and *T*_3_–*T*_0_, but also promoted imperceptible ∆WI_D_ in the three sessions (acceptable in the control). It is assumed that, in all these situations, the treatments promoted bleaching to such a degree that subsequent immersion in coffee was not capable of causing a variation in color and whiteness compatible with that observed in the control. This helps to explain the non‐progression of ∆*E*_00_ and ∆WI_D_ in WH among sessions and also the increase in whiteness promoted by OP and WHOP from *T*_1_–*T*_0_ to *T*_2_–*T*_0_ and *T*_3_–*T*_0_. Regarding hydrogen peroxide, a previous study [12] reported a reduction in color and whiteness variations, while another study [28] observed a reduction in color variations with just one bleaching session, but most likely because they used a higher concentration (HP 40%) than that of the present study. In contrast with our findings, a previous research [18] reported an increase in susceptibility to color variation, with a lower concentration of hydrogen peroxide but with a considerably longer exposure time (HP 6% 1 h/day for 10 days), which could confer greater potential for aggressiveness to the treatment. Despite this, whiteness was not adversely affected [18]. Regarding carbamide peroxide, although one study [18] used a higher concentration of this gel (CP 15% 6 h/day for 7 days), they also did not observe a significant effect on color and, as for whiteness, the findings were not clear. Another study [24] observed that, in the three sessions evaluated, there was an increase in susceptibility to color variation, which became perceptible. This divergence in results is probably related to the considerably longer exposure time (CP 10% 10 h/day for 14 days) used by the authors [24], making the material more susceptible to variations. The increase in susceptibility to color variation that occurred in the present study, only in the OP in *T*_3_–*T*_0_ in relation to *T*_1_–*T*_0_, was also observed in a previous study [24]. However, in addition to reporting earlier changes, the ∆*E*_00_ was acceptable in the second session and moderately unacceptable in the third session [27], while it was imperceptible in the present study, probably due to the lower exposure time. Still, whiteness was not adversely affected regardless of session [24]. On the contrary, after the second session, ∆WI_D_ went from acceptable in the control to imperceptible in the bleached group, which is in line with our results. In the present study, translucency was adversely affected by OP and WHOP when considering the thresholds (imperceptible in control and acceptable in both treatments) in *T*_3_–*T*_0_, indicating the influence of topographic changes resulting from both treatments on translucency, although there was no significant difference in relation to control. A previous study [24] reported adverse effects on the translucency of VE, with increased susceptibility to variations in this parameter in the three sessions, but with a much longer exposure time to the bleaching gel, justifying the more severe changes. Regarding hydrogen peroxide, in the present study, this bleaching gel had no effect on translucency and both the bleached and control groups presented imperceptible ∆RTP_00_, differing from a prior study [12], who reported a reduction in susceptibility to changes in translucency, with a change in ∆RTP_00_ from acceptable in the control to imperceptible in the bleaching treatment.

The distinct response of LU and VE materials can be related to their microstructural composition. Lava Ultimate, a resin nanoceramic, exhibits greater susceptibility to bleaching‐induced degradation and staining due to its higher polymer content. In contrast, VITA Enamic, with a predominant ceramic network, demonstrated greater resistance to degradation, maintaining color stability after bleaching and staining.

Considering the findings of the present study, it can be stated that the proposed bleaching treatments performed well in the three sessions, without harming the esthetic outcomes of the materials. The main limitation of the present study is the absence of saliva on the surface of the materials during at‐home bleaching, differing from the clinical scenario. Although the present study indicates that, from an esthetic point of view, the bleaching treatments could be recommended, studies that evaluate biomechanical properties are necessary, considering the topographic changes observed in both materials.

## 5 Conclusions

Within the limitations of this current study and based on the findings, the following conclusions were drawn. The distinct bleaching treatments and successive bleaching sessions followed by staining influenced the changes in color, whiteness, and translucency of both materials. Although bleaching with any of the tested protocols changed the optical properties, the mean values were considered imperceptible or deemed acceptable.

## Funding

This work was supported by the Fundação de Amparo à Pesquisa do Estado de São Paulo (Grant 2021/04476‐6).

## Conflicts of Interest

The authors declare no conflicts of interest.

## Data Availability

The data that support the findings of this study are available from the corresponding author upon reasonable request.
